# A Systematic Review of Lean Implementation in Hospitals: Impact on Efficiency, Quality, Cost, and Satisfaction

**DOI:** 10.34172/ijhpm.8974

**Published:** 2025-08-26

**Authors:** Jingjing Wang, Hui Lv, Mingxin Chen, Chenyang Liu, Wenjie Ren, Hui Jiang, Lizhang Zhang

**Affiliations:** ^1^Institutes of Health Central Plains, Henan Medical University, Xinxiang, China.; ^2^The Second Affiliated Hospital of Henan Medical University, Xinxiang, China.; ^3^The First Affiliated Hospital of Henan Medical University, Xinxiang, China.; ^4^School of Public Health, Henan Medical University, Xinxiang, China.

**Keywords:** Lean Healthcare, Lean Management, Lean Implementation, Hospitals

## Abstract

**Background:**

Lean healthcare practices are widely used to enhance efficiency, quality, cost-effectiveness, and satisfaction in hospitals. However, no studies have synthesized their effectiveness across these dimensions. This review aims to address this gap by evaluating the impact of Lean implementation on four key themes: Efficiency, quality, cost, and satisfaction.

**Methods:**

Four online databases were selected for the targeted articles: Scopus, Medline, PubMed, and Web of Science. Additionally, a comprehensive search was conducted using the Google Search Engine, along with a review of the citation list from the retrieved articles, to identify related grey literature and acquire additional articles. The search covered only the period from January 2019 to October 2024. The quality and research methodology of the articles reviewed were evaluated to determine the reliability of these findings.

**Results:**

A total of 6021 articles were screened, and 60 were included in this study. Our findings were grouped into four themes: (1) Efficiency: 49 studies identified 12 sub-dimensions of efficiency, with the most common variables being waiting time, length of stay (LOS), and patient volumes. (2) Quality: 12 studies reported quality improvements, covering 12 variables, with 30-day readmission rates, counselling sessions, and drug-related indicators being most prominent. (3) Cost: 17 studies examined Lean-driven cost reductions, with operating costs being the most frequently addressed variable, appearing in seven studies. (4) Satisfaction: Key satisfaction indicators included patient satisfaction, Hospital Consumer Assessment of Healthcare Providers and Systems (HCAHPS) scores, complaint rates, and nurse satisfaction.

**Conclusion:**

This is the first review to synthesize the literature on the impacts of Lean implementation across four key themes, while also identifying existing gaps. It highlights the positive outcomes of Lean in hospitals and outlines the primary areas of improvement emphasized by healthcare institutions within each theme.

## Background

 The intensified competitiveness within the healthcare industry has compelled hospitals to prioritize continuous improvements in quality and efficiency as key development goals,^1,2^ driving them to adopt management models focused on lean practices. These models are seen as crucial for ensuring long-term sustainability in the face of future market challenges. As advocated by researchers,^3-5^ effective hospital management necessitates the adoption of systematic concepts and methodologies aimed at facilitating comprehensive reforms, enabling hospitals to achieve substantive progress. However, identifying comprehensive systems theories and evidence-based approaches can be challenging in practical settings, while the introduction of Lean within the medical industry has been effectively addressing this issue. Lean is a well-established, evidence-based methodology that has demonstrated its potential to assist hospital managers in enhancing hospital management and achieving favorable outcomes.^3^ Since its introduction to the healthcare sector, the practice of lean principles, thinking, and tools has provided hospitals with notable benefits.^4-6^

 Lean management, originally developed by Toyota in Japan during the 1950s, has since evolved in the business and manufacturing sectors and was later adapted and introduced into the healthcare industry.^7^ As early as 1995, Joan Wellman, a real pioneer in the field of lean healthcare, took the initiative to collaborate with a hospital in Seattle on Lean work.^8^ In 2001, the efforts to integrate Lean in healthcare were initiated in the UK.^9^ In 2002, the Virginia Mason Medical Center in the United States initiated the introduction and implementation of Lean management.^10^ In recent years, it has been widely adopted and implemented in hospitals across other various countries, such as China,^4^ Italy,^5^ Spain,^11^ Brazil,^12^ Japan,^13^ and Netherlands.^14^ Lean management has gained popularity in healthcare due to its focus on eliminating waste, optimizing processes, and enhancing value.^2,4,9^ As Bicheno mentioned, by emphasizing the minimization of process inefficiencies and the maximization of value-added, organizations can enhance their performance regarding cost, quality, and time.^14^ In the context of healthcare, the principle of ‘respect for people’ remains a paramount success factor in the effective implementation of Lean methodologies.^6^ This principle, serves as the foundation for continuous improvement, involves investing in employees, in training, job security, and their morale.^15^

 Various researchers have examined the positive effects of lean implementation in healthcare settings from multiple perspectives.^5,9,12^ Efficiency improvement, particularly in terms of reducing time and optimizing processes, is the most frequently addressed topic in the application of Lean in hospitals and is also the most extensively studied by scholars. AlHarthy et al reported a significant reduction in the proportion of patients discharged without scheduled follow-up appointments following the implementation of lean practices in oncology settings.^16^ Pellini et al suggested that lean management practices could improve both preoperative and postoperative processes amid the ongoing pandemic, thereby optimizing the utilization of limited resources and enhancing efficiency through better time management.^17^ Muharam and Firman found that the adoption of lean principles in* in vitro *fertilization treatment led to a shortening of total patient wait time and an increase in the value-added ratio.^18^

 Quality, cost and satisfaction are also key focal points for scholars studying Lean implementation in healthcare. Ayaad et al discovered that the application of lean management significantly enhanced service quality, cost control, and efficient time management in oncology settings.^19^ Similarly, Kurnia et al observed improvements in customer satisfaction, evidenced by a 44.5% reduction in the number of complaints, alongside a 34.2% decrease in the lead time for medical device procurement.^20^ An integrated review of Lean healthcare in 2023 highlighted the potential of Lean methods to significantly decrease the length of hospital stays for patients and the reductions in hospitalization-related costs.^21^ Tillmann et al enhanced their organization’s core competitiveness by applying lean management to develop their supply chain management system.^22^ This approach improved the integration of supply chain functions, which, in turn, led to enhanced performance. Since the implementation of lean management in American hospitals began earlier, there has been a greater body of system-level research on its effectiveness in healthcare settings. For instance, Rundall et al conducted a nationwide survey of 1152 US hospitals to explore the relationship between lean management and hospital performance.^23^ Similarly, Po et al examined the relationship between lean management and hospital performance by surveying 288 US public hospitals, indicating that lean management was linked to the EBITDA (Earnings before interest, taxes, depreciation, and amortization) and the percentage of patients leaving the emergency department (ED) without being seen.^24^ Overall, while most existing research on lean management has focused on individual lean projects or departments, studies examining comprehensive lean management systems are relatively limited, with much of the research concentrated in developed countries such as the United States.

 While many studies have highlighted the positive outcomes associated with lean implementation in healthcare, not all findings have been uniformly successful.^25,26^ One example is a study conducted in Sweden, which found that care centers adopting lean did not demonstrate a statistically significant improvement in patient satisfaction throughout the period.^27^ Kunnen et al identified several barriers to sustaining lean management in healthcare and classified them into key factors, including the overburdening of employees with additional responsibilities, insufficient staff involvement, patient engagement, resources for engagement, leadership commitment, and adequate follow-up on projects.^28^ These mixed results underscore the need for further investigation into the factors influencing the success of lean management in healthcare contexts.

 Previous reviews have primarily focused on identifying which Lean tools have been applied in healthcare,^21,26,29^ determining the types of waste that should be prioritized for elimination in hospitals, or summarizing the structural frameworks of Lean. Lean implementation is often characterized by a time-bound cycle and, in many hospitals, is applied through specific projects rather than across the entire organization. As a result, existing research frequently struggles to capture the long-term, overarching effects of Lean. Even when the benefits of Lean are acknowledged, few studies explore these four dimensions—quality, efficiency, cost, and satisfaction—holistically to identify actionable implementation strategies. This gap underscores the need for further research that adopts a broader and more cohesive approach to Lean implementation. To the best of our knowledge, this article is the first to provide a comprehensive interpretation of the effects of Lean implementation in hospitals from an integrated perspective. This approach offers a more nuanced understanding of how Lean can drive hospital development across multiple dimensions. The primary objective of this review is to systematically assess the impact of Lean implementation in hospitals across these four key dimensions: Quality, efficiency, cost, and satisfaction. By doing so, the review aims to provide practical recommendations for practitioners involved in hospital management. Consequently, the main research question for this review is as follows: How has the application of Lean in hospitals contributed to improvements in efficiency, quality, cost, and satisfaction?

## Methods

###  The Conceptual Framework for Lean Implementation Effectiveness in Hospitals

**Figure 1:**
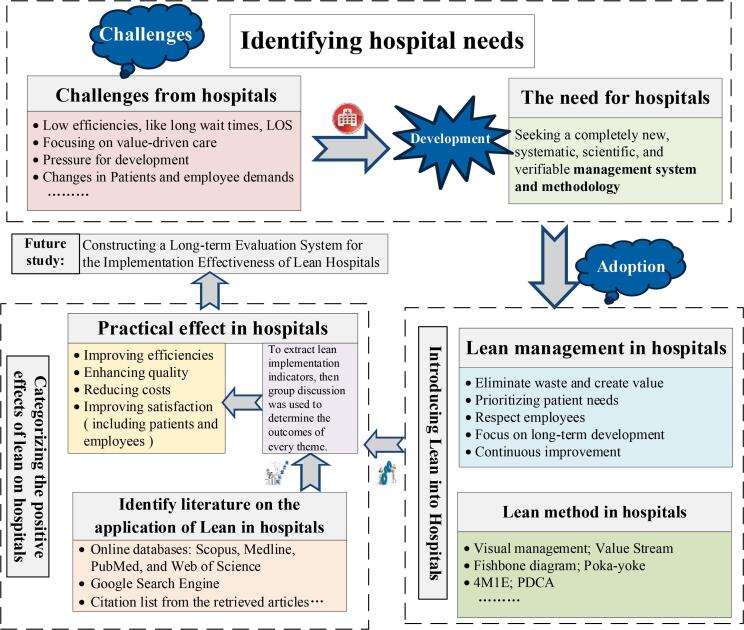
The Conceptual Framework for Lean Implementation Effectiveness in Hospitals. Abbreviations: LOS, length of stay; PDCA, Plan-Do-Check-Act. Note: 4M1E is one of the lean tools, covering four main elements and one environmental factor: ‘4M’ refers to Man, Machine, Material, and Method; ‘1E’ stands for Environment.

 To clarify and define the entire research framework more explicitly, we construct a conceptual framework diagram for presentation (Figure 1). Specifically, the challenges currently faced by hospitals were identified, highlighting the need for a new, systematic, scientific, and verifiable management system and approach to address these difficulties and support hospital development. This approach should be applied and tailored to the specific context of each hospital, thereby guiding them toward an internally driven, high-quality, and innovative development path. Following this, the value of introducing Lean methodologies into hospitals was shown. Both the management and methods have been shown to effectively contribute to improvements within hospital. Subsequently, diversifying methods were utilized to identify and select relevant articles. Then, data extraction was performed from the articles that met the established criteria, and the practical outcomes of Lean application in hospitals were visualized across four key dimensions: Efficiency, quality, cost, and satisfaction. Additionally, we intend to explore the development of a long-term evaluation system to assess the effectiveness of Lean Hospital implementation in future research. This system will serve to enhance the sustainability and stability of Lean practices within hospitals, facilitate the integration of Lean into hospital culture, and assist the long-term development of healthcare institutions.

###  Literature Screening Process

**Figure 2:**
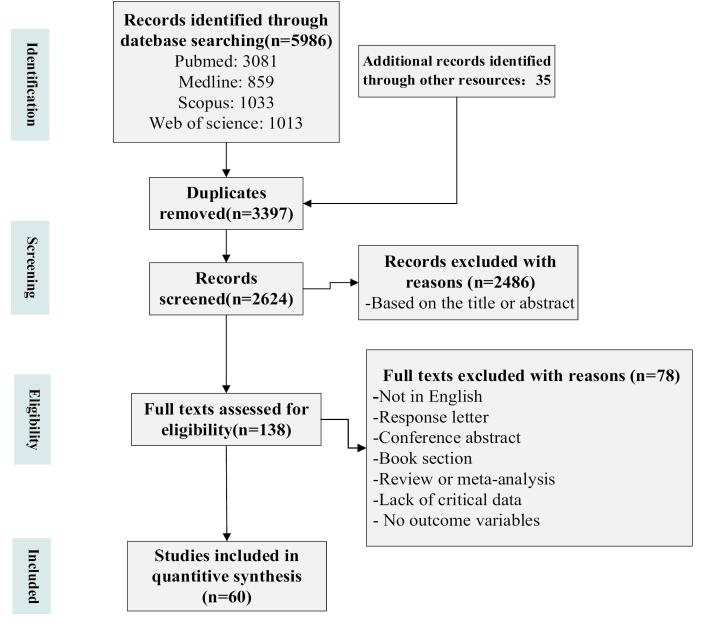
The PRISMA Flowchart. Abbreviation: PRISMA, Preferred Reporting Items for Systematic Reviews and Meta-Analyses.

 The methodology employed for the literature screening in this review was a systematic literature review.^30,31^ The literature screening process strictly followed the guidelines established by the Preferred Reporting Items for Systematic Reviews and Meta-Analyses (PRISMA).^32^ The whole selection process was illustrated in Figure 2. The subsequent subsections provided a comprehensive presentation of the method employed.

###  Data Source and Search Strategy

 Four online databases were selected for the targeted articles: Scopus, Medline, PubMed, and Web of Science. Additionally, a comprehensive search was conducted using the Google Search Engine, along with a review of the citation lists from the retrieved articles, to identify related grey literature and acquire additional articles. The search covered only the period from January 2019 to October 2024. Concurrently, a preliminary search was undertaken to develop an effective search procedure in line with the Peer Review of Electronic Search Strategies guidelines. The terms of search were identified by the following keywords: “Lean management,” “Lean principles,” “Lean thinking,” “Lean approach,” “Hospital,” “Healthcare sector,” “Quality,” “Efficiency,” “Benefits,” and “Satisfaction.” The specific search strategy employed was detailed in Supplementary file 1.

###  Participants 

 Studies of healthcare units included general hospitals, specialized hospitals, clinics, teaching hospitals or health centers, and all these hospitals had conducted projects for acquiring improvement on some aspects. This study established specific inclusion and exclusion criteria to facilitate the selection of appropriate articles. Specifically, the following inclusion criteria were applied in this study: (1) Peer review articles; (2) The application of lean within healthcare settings; and (3) Having comparative outcomes of lean practice. The following exclusion criteria were delineated in this study: (1) Not in English; (2) Response letter; (3) Conference abstract; (4) Book section; (5) Review or meta-analysis; (6) Lack of critical data; and (7) No outcome variables.

###  Data Extraction, Analysis and Synthesis

 We implemented rigorous screening procedures to identify eligible articles for inclusion in this review. Initially, two independent reviewers (JW and HL) assessed each study based on its title and abstract. Subsequently, the reviewers checked the entire texts of the relevant articles according to the established inclusion and exclusion criteria. In cases where the reviewers could not come to an agreement, a third reviewer (MC) was brought in to facilitate discussion and resolve any disagreements. All reviewers participating in this study have rich experience and knowledge in lean healthcare, and some of them have published some studies related lean healthcare.

 We developed a standardized data extraction table through group discussions, which included key information such as the title, first author(s) names, publication year, country, journal, study design, statistical tests, and outcome variables. Two data extractors (JW and HL) were then designated to extract the data, and once the extraction was completed, the consistency of the extracted data was checked by comparing the results. Any discrepancies were resolved through discussion, with a third team member (HJ) joining if necessary to reach consensus. Subsequently, we classified the information into four dimensions based on the extracted data: Efficiency, quality, cost, and satisfaction, through further group discussions based on the sampled articles. Finally, additional discussions were conducted to determine how to consolidate and synthesize the data within each dimension, and the results were presented in a table format. Considering the heterogeneity of researches in terms of their study designs, and outcomes, we were unable to pool the results and conduct a meta-analysis. As a result, we decided to conduct a descriptive synthesis of the outcomes to summarize findings in these articles included, as in similar surverys.^2,6^

###  Risk of Bias

 We utilized the quality assessment tool developed by Hawker et al^33^ for assessing the quality of these targeted articles. The tool comprises nine key attributes: Abstract and title; introduction and aims; method and data; sampling; data analysis; ethics and bias; findings/results; transferability/generalizability; and, implications and usefulness.^33^ Each attribute is rated on a four-point scale: good (4 points), fair (3 points), poor (2 points), and very poor (1 point). The final quality rating of each article is determined by summing the scores for all attributes. The quality ratings for the included articles in this review followed the classification proposed by Braithwaite et al^34^ which categorizes articles into three quality levels based on the total score: high (30-36 points), medium (23-29 points), and low (9-22 points). Two independent reviewers assessed the included articles for ensuring the scientific rigor and validity of the evaluation. In cases of disagreement regarding the quality rating of an article, a third reviewer was consulted to facilitate discussion and reach a consensus. The outcomes of assessment were depicted in Supplementary file 2. A narrative synthesis of the findings was shown in the part of results.

## Results

 Initially, a total of 6021 articles were identified through four online databases, as well as Google searches and references from related literature. Subsequently, 3397 articles were excluded due to duplication. After that, 2486 articles were removed based on the title and abstract in the screening stage. A full-text review was then conducted for 138 articles, resulting in the exclusion of 78 articles for reasons detailed in Figure 1. Ultimately, 60 articles were deemed suitable for inclusion in this review.

###  Risk of Bias and Quality Assessment 

 Given the variation in the research designs of the targeted studies, the Hawker et al^33^ quality assessment tool was deemed an appropriate assessment tool. The articles were categorized into three quality levels: High, medium, and low. The distribution of articles across these categories was 41.7%, 50%, and 8.3%, respectively. Detailed quality scores were shown in Supplementary file 2.

###  Basic Information for Article Included in This Review 

**Figure 3:**
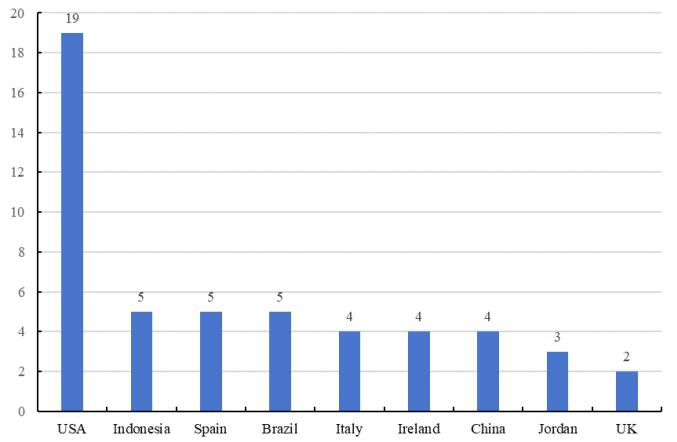
The Top Nine Countries by Article Included in This Review.

**Figure 4:**
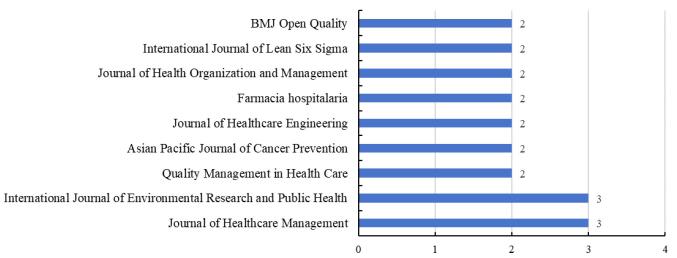
The Top Nine Journals by Article Included in This Review.

 There were 60 studies assessing the implementation of lean in hospitals met the predefined inclusion criteria. We found that these studies were carried out in various countries (n = 19), with the United States representing the largest proportion, accounting for 30.6% (n = 19). 53.3% of the studies were conducted in Indonesia (n = 5), Spain (n = 5), Brazil (n = 5), Italy (n = 4), Ireland (n = 4), China (n = 4), Jordan (n = 3) and UK (n = 2). It has been shown in Figure 3. We also found that these articles were mainly published in management-related journals, with the top two journals being “*Journal of Healthcare Management*” and “*International Journal of Environmental Research and Public Health*,” as shown in Figure 4. Twelve studies evaluated the effectiveness of lean implementations across multiple hospitals in this review, while the remaining studies focused on lean improvement projects within a single hospital.

###  Theme Classification 

 Lean, with its emphasis on maximizing benefits while minimizing investment, has gained widespread adoption among hospital managers since its introduction to the healthcare sector. This research primarily explored the impact of Lean implementation on hospitals, based on four key themes identified through narrative analysis. The 60 included articles were categorized into these four themes—Efficiency, Quality, Cost, and Satisfaction—based on the outcome variables assessed in each study.

###  Theme 1: Efficiency 

 Efficiency is the most frequently cited outcome in the context of Lean effectiveness, and it constituted the first theme covered in this review. In this study, efficiency refers to the systematic identification and elimination of non-value-added waste activities through the application of lean principles and tools, aimed at optimizing workflows to maximize the effective use of resources and time management, ultimately enhancing overall operational effectiveness. We systematically reviewed the included studies and summarized the variables associated with efficiency (n = 49), integrating a total of 12 sub-dimensions commonly used by researchers to assess Lean efficiency or the areas often targeted for improvement through Lean. We found that the majority of hospitals implementing Lean management were public hospitals, with only five being private hospitals. The size of sample hospitals included large, medium, and small, with the majority being large hospitals. Only six hospitals were medium-sized, and one was small. Regarding departments, Lean management was more commonly applied in single-department studies, with the top four departments being surgery, pharmacy, emergency, and operating rooms. The three most frequently mentioned variables are: waiting time, length of stay (LOS), and patient volumes.

 Waiting time was identified as the primary issue across the studies, with 12 articles including it. Obviously, it has suggested that waiting time was the most commonly used indicator for efficiency improvement. There were variations in the extent of waiting time reductions across these studies, ranging from 11.3%^5^ to 88.03%.^35^ For instance, Catherine et al reported a 60% improvement in waiting times,^36^ while Martins and Sérgio observed a 53.8% reduction.^37^ Specific details on waiting time reductions were also provided in several studies. Reis et al reported a decrease in bed hygiene waiting time from 13.45 hours to 1.61 hours, representing an 88.03% reduction.^35^ Hammoudeh et al found significant reductions in patient waiting times for prescriptions, with waiting times for prescriptions of fewer than three medications decreasing from 22.3 minutes to 8.1 minutes (63.7%), and for prescriptions of three or more medications, from 31.8 minutes to 16.1 minutes (49.4%).^38^ Additionally, Ullah et al reported a 26% improvement in the percentage of medical reviews conducted by a doctor within 15 minutes, up from 0%.^39^

 LOS is another critical indicator of efficiency improvement following the implementation of lean in hospitals. In this review, nine articles reported reductions in LOS, with four studies indicating a significant relationship between lean adoption and LOS reduction.^24,40,41^ Additionally, five studies reported a decrease in LOS as a result of lean application. For example, Fiorillo et al found that the average preoperative LOS represented a 22.4% reduction.^42^ Similarly, Alexander et al reported that psychiatric patients experienced a shorter LOS in the ED, from 8 hours to 7 hours, a 12.5% decrease.^7^

 Upon pooling the targeted studies in this review, we found that six studies reported improvements in patient volumes following Lean implementation, with increases ranging from 15%^11^ to 65%.^5^ Additionally, we identified five studies that investigated Lean improvements in patient discharged. Of these, three studies explicitly demonstrated that Lean was effective in reducing patient discharge time.^16,40,43,44^ One study, in particular, revealed a significant association between Lean adoption and a decrease in the proportion of patients leaving the ED without being seen, further supporting Lean’s effectiveness.^40^

 We also reviewed five studies that assessed the impact of Lean on lead time.^20,37,45-47^ All of these studies reported a significant reduction in lead time following the implementation of Lean initiatives. Notably, one study reported a substantial decrease in expected lead time, from 222 minutes to 42 minutes, representing an 81.1% reduction.^47^

 Furthermore, five studies focused on improvements in the time related-equipment,^48-52^ while four studies examined enhancements in process time.^52-55^ Other related categories were summarized in Table 1.

**Table 1:** The Effect of Lean Applications in Hospitals on Efficiency

**Codes**	**Hospital Type**	**Hospital Size**	**Departments**	**Indicators**	**Values**
Waiting time	—	Large	Internal medicine, family medicine, and pediatric	Patient wait time^56^	-1.2% per month
	Public	Large	Fertility clinic	Total patient waits time^18^	-51.90%
	—	Large	Pharmacy	Waiting times significantly improved^57^	-30.50%
	Public	Large	Operating room	Waiting times for operations^58^	-35.00%
	Public	Large	Surgery	The time the bed waited for hygiene^35^	-88.03%
	—	Medium	—	Waiting time for patients^37^	Approximately 53.8%
	Private	Small	Outpatient	The average total waiting time^59^	-37.92%
	Public	Large	ED	The percentage of waiting for counseling^5^	-11.30%
	Public	Large	ED	Waiting times for medical review by a doctor < 15 min and analgesia waiting times of 16–30 min	-26%; -22%
	Public	Medium	—	The waiting time for vaccination^60^	-25.12%
	Private	Large	Pharmacy	Patient waiting time for prescriptions of fewer than 3 medications and of 3 medications or more^38^	-63.68%; -49.37%
	Public	Large	Diagnostic imaging, surgery, pathology and the cancer centre	Wait time^36^	-60%
LOS	Public	—	Whole hospital	Lower severity-adjusted geometric LOS showed significant bivariate relationships with Lean adoption^24,40^	*P* < .05
	Public	Large	ED	LOS^61^	-6.67%
	Public and private	—	Whole hospital	ALOS are significantly correlated with lean^41^	b = -0.356, *P* < .01
	Public	Large	Surgery	Average LOS^43^	-14.41%
	Public	Large	Surgery	The average length of hospital stays in new pre-admission area^62^	-78%
	Public	Large	Maxillofacial surgery	The average preoperative LOS^42^	-22.40%
	Public	All	Whole hospital	The adoption of Lean IT was significantly associated with a reduction in severity-adjusted geometric LOS^35^	b = -0.098, *P* = .018
	Public	Large	ED	LOS of psychiatric patients in the ED; LOS exceeded 24 h^12^	-12.5%; -3%
Patient volumes	Public	Large	Surgery	2014 and 2018 the number of patients on the SWL^62^	23.50%
	Public	Large	Surgery	Surgical capacity without the need for new resources^11^	15%
	Public	Large	Emergency room	Occupancy rate^63^	43.10%
	Public	Large	Surgery	The capacity of patient admissions^12^	20.75%
	Public	Large	—	The number of patients treated^5^	2799 in 2018 to 8979 in 2021
	Public	Large	Diagnostic imaging, surgery, pathology and the cancer centre	Monthly patient volumes^36^	65%
Patients discharged	Public	All	Whole hospital	Lean adoption was significantly associated in the direction predicted with the percentage of patients leaving the ED without being seen^40^	b = -0.610, *P* < .068
	—	Large	Surgery	The average time between computer entry of discharge orders and patient’s departure from the unit^43^	-30%
	Public	Large	Ophthalmology	The mean time between registration and discharge of a patient^44^	240 ± 14.14 to 60 ± 8.16 min
			Acute care ward	The outcome metric prenoon discharges on both wards^64^	16% on ward X; 10% on ward Y
	Public	Large	Inpatient	The percentage of patients discharged without planned follow-up appointments^16^	-9%
Lead time	Private	Medium	Surgery	The ratio of productive time to lead time during morning rounds was higher after lean adaptation^45^	3.73 vs. 2.03
	Private	Large	Pharmacy	Lead time in scenario-1 and in scenario-2^46^	-93.27% and -94.46%
	Public	—	Warehouse and logistics	The lead time for the procurement of medical devices^20^	-34.20%
	Public	—	Ear, nose, and throat; audiology; neuroradiology	Lead time^47^	-81.08%
Process time	Public	—	Orthopedic surgery; general surgery; otorhinolaryngology	The overall scheduling time and time reductions in the revised process^53^	-13.29%; -37.37%
	Public	Large	Oncology	The average time for the closure of reported medication incidents and non-medication incidents; maximum closure days for the medication group and the non-medication group^54^	-44.78%; -54.48%; -86%; -68%
	Public	Large	Pharmacy	The inpatient medication returns process^52^	-67%
	Public	Large	Pharmacy	The average process time^55^	-18.27%
Patient referrals	Public	Medium	Tobacco treatment center	New patient referrals^65^	140.00%
	Public	—	Ear, nose, and throat; audiology; neuroradiology	Referral to treatment time^47^	-69.78%
	Public	Large	Inpatient	Patient transfers decreased^66^	-30% and 23% in terms of total distance and transfer episodes, respectively
Operating room time	Public	Large	Otolaryngology–head and neck surgery	Operating room time^67^	-10.82%
	Private	Large	Surgery	Operating room turnover time^68^	-6.22%
Room turnover/turnaround times	Private	Large	Infection prevention & control	Median room turnover; mean turnover time in between patients^69^	-50%; (10 ± 1.41 to 8 ± 2 min)
	Public	Large	Clinical laboratory	Turnaround times in the delivery of glucose test results at the adult emergency service^70^	-13.10%
The time related-equipment	Public	Large	Radiology	The percentage of CT scans overdue for scheduling^48^	-16.03%
	Public	Medium	Operating room	The time taken to count each surgical tray and the average overall instrument utilization rate^49^	-40%; -24%
	Public	Large	Ward treatment room	The mean time taken to gather equipment for IVC and the need for house officers to ask other ward staff for help to locate equipment^50^	-64.28%; -62.3%
	Public	Large	Pharmacy	Medication label printing time^52^	-70%
	Public	Large	Operating room	The use of chest X-rays and cardiac visits^51^	-27%; -12%
Inventory	Public	Large	Whole hospital	The inventory time of the warehouse in each ward and materials in the treatment room during shift hand-over^71^	Decreased
	Public	Large	Pharmacy	Inventory management efficiency^52^	36%
Others	Public	Large	ED	30-day acute care utilization^61^	0.30%
	Private	Large	Infection prevention & control	The duration of room cleaning and curtain changing^69^	-35.09%
	Private	Medium	ED	D2N time^72^	Reduced by 36 min
	Public	Large	Radiology	Within 60 min of patients' arrival in a pre- and post-procedure care area^73^	35%
	Public	—	Outpatient	The work efficiency of senior doctors and the patient flow of associate senior doctors^74^	25%; 50%
	Public	—	Whole hospital	Lean practices are positively and significantly associated with healthcare operational performance^75^	*P* < .001
	Public	Large	Operating room	No value-added time^51^	-9%

Abbreviations: LOS, length of stay; ED, emergency department; IT, information technology; CT, computed tomography; SWL, Surgical Waiting List; D2N, door-to-needle; IVC, intravenous cannulation; ALOS, average length of stay. Notes: “—” means that there was no related information in the targeted articles. “All” means that hospital size included large, medium, and small. “-” means “negative sign.”

###  Theme 2: Quality

 Lean implementation in hospitals has been widely recognized for its potential to enhance quality, as noted by numerous scholars. However, most existing research primarily assessed the impact of Lean on quality improvement through single in-hospital projects or cross-sectional studies, with limited attention given to a systematic perspective on which specific aspects of quality can be enhanced through Lean. In this review, we identified 12 studies that reported improvements in quality following Lean implementation, which encompassed 12 distinct variables. The hospital types involved in the quality theme primarily included public hospitals and private hospitals, with eight studies conducted in public hospitals and only two in private hospitals. Six studies were conducted in large hospitals, with only one in a medium-sized hospital. We found that the majority of studies were conducted at the hospital level. Single-department studies were rare and included departments such as pharmacy, emergency, and oncology.

 Among these, the top three variables were the 30-day readmission rate, counseling sessions, and drug-related indicators. Specifically, three studies explicitly found a significant correlation between Lean implementation and reductions in 30-day readmission rates,^23,40,61,76^ while another study reported a decrease in the 30-day readmission rate, from 21% to 19.3%, following the implementation of Lean.^61^ Three studies examined the impact of Lean on counseling sessions and reported that Lean resulted in an increase in the time spent between patients and doctors, with improvements ranging from 13%^65^ to 23.3%.^52^ Two studies focused on drug-related variables, including the number of available drug dosage forms and the number of high-risk drugs, which decreased by 56.72% and 40.73%, respectively.^77^

 Further studies reported improvements in other quality indicators, such as a reduction in the rate of pressure ulcers,^23^ low-mortality diagnosis-related groups (DRGs),^40^ enhanced patient safety,^40^ and improvements in the timeliness of care.^40^ We also identified a significant correlation between Lean implementation and appropriate use of medical imaging,^40^ electronic health record (EHR)-based decision support,^78^ and the use of quality-focused information management.^78^ Moreover, several studies found that Lean implementation led to a notable reduction in adverse events^71^ and clinical defects,^79^ as well as improvements in service quality^19^, as summarized in Table 2.

**Table 2:** The Effect of Lean Applications in Hospitals on Quality

**Codes**	**Hospital Type**	**Hospital Size**	**Departments**	**Indicators**	**Values**
Lower 30-day unplanned readmission rate	Public and private	All	Whole hospital	Lower 30-day unplanned readmission rate^23^	b = -0.066, *P* =.051
	Public and private	All	Whole hospital	The degree of Lean implementation was associated with lower 30-day unplanned readmission rate^76^	b = -0.01, *P* <.007
	Public	All	Whole hospital	Lower 30-day readmission rates showed significant bivariate relationships with Lean adoption^40^	b = -0.053, *P* =.001
	Public	Large	ED	30-Day readmission rates^61^	-1.70%
Counseling sessions	Public	Medium	Tobacco treatment	Mean counseling sessions^65^	13%
	Public	—	Ear, nose, and throat	Patient contact time^47^	18.06%
	Public	Large	Pharmacy	Patient counseling time^52^	23.30%
Drugs-related	Public	Large	Inpatient	The number of pharmaceutical dosages forms available and the number of high-risk drugs^77^	-56.72%; -40.73%
	Public	Large	Pharmacy	Medication expiry checks and prescription verification^52^	200%, 50%
Lower pressure ulcer rate	Public and private	All	Whole hospital	Lower pressure ulcer rate^23^	b = -0.0001, *P* =.071
Lower death rates in low-mortality DRGs	Public	Large	Whole hospital	Lower death rates in low-mortality DRGs showed significant bivariate relationships with Lean adoption^40^	*P* =.002
Patient safety	Public	Large	Whole hospital	Patient safety showed significant bivariate relationships with Lean adoption^40^	*P* <.001
Timeliness of care	Public	Large	Whole hospital	Timeliness of care showed significant bivariate relationships with Lean adoption, and the adoption of Lean in public hospitals was significantly associated with timeliness of care^40^	*P* <.001
Appropriate use of medical imaging	Public	Large	Whole hospital	The adoption of Lean in public hospitals was significantly associated with better-than-average national performance on the appropriate use of medical imaging^40^	*P* <.001; b = 0.097, *P* =.007
EHR-based decision support	Public and private	All	Whole hospital	The number of years doing Lean was positively associated with use of EHR-based decision support^78^	β = 0.011, *P* =.045
Use of quality-focused information management	Public and private	All	Whole hospital	The number of years doing Lean was positively associated with use of quality-focused information management^78^	β = 0.010, *P* =.045
Adverse events	Public	Large	Whole hospital	Incidence of nursing adverse events^71^	-2%, *P* <.05
Quality of services	Private	Large	Oncology	Quality of services^19^	3.84 ± 0.56, β = 0.512, *P* <.001
Clinical defects	Private	Large	Whole hospital	Clinical defects^79^	-2.80%

Abbreviations: EHR, electronic health record; DRGs, diagnosis-related groups; ED, emergency department. Notes: “—” means that there was no related information in the targeted articles. “All” means that hospital size included large, medium, and small. “-” means “negative sign.”

###  Theme 3: Cost

 Cost reduction is one of the most frequently cited benefits of Lean implementation, particularly for hospital managers seeking to control expenses and alleviate the operational burden on healthcare institutions. Cost refers to the financial resources spent by healthcare institutions in the context of Lean healthcare implementation in this study, categorized into direct costs (eg, operational costs) and indirect costs (eg, opportunity costs and savings from efficiency improvements). In this review, we identified 17 studies that focused on Lean-driven cost reductions, encompassing a total of nine distinct variables used to assess cost-saving outcome variables. In terms of hospital characteristics, Lean management was less frequently applied in private and small-sized hospitals. Specifically, only two studies were conducted in private hospitals, and two studies were conducted in small-sized hospitals. Additionally, we found that Lean management was applied in single departments, with the most common departments being operating rooms (2 studies), surgery (1 study), emergency (1 study), pharmacy (1 study), and fertility clinics (1 study).

 The most commonly examined variable across these studies was operating costs, which were addressed in seven of the studies. Multiple studies have highlighted the effectiveness of Lean implementation in reducing hospital costs. Specifically, three studies reported reductions in operating costs, with savings ranging from 8.7%^79^ to 80%.^26^ Additionally, four studies provided direct cost savings, such as one study showing a total cost savings of €1178.90^49^ and another conducted in the United States reporting average savings of US$ 400 000 through Lean implementation.^80^

 Another frequently examined variable was Medicare spending per beneficiary/inpatient expense per admission, with all five studies in this category originating from the United States.^23,40,35,72,76^ One study found a positive correlation between Lean implementation and Medicare spending,^23^ while three studies reported a similar correlation between Lean implementation and inpatient expense per discharge.^35,40,76^ Notably, one of these studies also observed a negative correlation between the application of visual management tools and adjusted inpatient expense per discharge.^35^ Three studies focused on the EBITDA margin, all of which were conducted in the United States, and all indicated a proportional relationship between Lean implementation and improvements in the EBITDA margin.^24,35,40^ Medicine of cost was another area where Lean implementation showed cost-saving effects. One study reported a reduction of US$ 22 097 in medical costs over a three-month period following Lean implementation.^81^ Additionally, one study observed a decrease in the cost of sterilizing pediatric minor set, dropping from €60 to €49.52, a reduction of 17.5%.^49^ Furthermore, two studies related to inventory optimization, along with one each involving participation in bundled payment schemes,^35^ net profit margin (NPMAR),^41^ value-added rate,^18^ and rapid improvement events (RIE),^79^ all demonstrated the cost-saving benefits of Lean implementation, as summarized in Table 3.

**Table 3:** The Effect of Lean Applications in Hospitals on Cost

**Codes**	**Hospital Type**	**Hospital Size**	**Departments**	**Indicators**	**Values**
Operation costs/cost savings	Public and private	All	Whole hospital	Operating margin are significantly correlated with lean^41^	b = 0.005, *P* < .10
	Public	Large	Surgery	4-Year project in operation costs saving^62^	EUR 25.5 million
	Public and private	Large	Operating room	Operation costs savings^80^	Annual of US$ 400 000
	Public	Medium	Operating room	The total cost savings^49^	€1178.90
	—	Medium	Whole hospital	Operational cost saving^26^	80%
	Public	—	Ear, nose, and throat	Costs saving^40^	Saving £5.9 million per year
	Private	Large	Whole hospital	Real cost savings and real dollar cost savings in EDs than in other settings^79^	28.8%; 8.7%
Medicare spending per beneficiary/inpatient expense per admission	Public and private	All	Whole hospital	Adopting Lean was significantly associated with lower Medicare spending per beneficiary^23^	b = −0.005, *P* = .027
	Public and private	All	Whole hospital	The degree of Lean implementation measured was associated with lower adjusted inpatient expense per admission^76^	b = -38.67; *P* < .001
	Public	All	Whole hospital	The adoption of Lean in public hospitals was significantly associated with lower adjusted inpatient expense per discharge^40^	b = -0.203, *P* = .045
	Public	All	Whole hospital	Lean IT adoption was associated with adjusted inpatient expense per discharge and visual management tools were also associated with lower adjusted inpatient expense per discharge^35^	b = -0.112, *P* = .090; b = -0.176, *P* = .034
	Private	Medium	ED	The conservation of per patient^72^	Mean of 68.4 million neurons
EBITDA	Public	All	Whole hospital	Lean adoption was significantly associated in the direction predicted with EBITDA^24^	b = 0.042, *P* < .020
	Public	All	Whole hospital	Lean adoption in public hospitals was marginally associated with a higher EBITDA margin^40^	b = 0.114, *P* = .055
	Public	All	Whole hospital	Lean IT adoption was found to be significantly related to EBITDA margin^35^	b = 0.077, *P* = .077
Medicine of cost	Public	Small	Pharmacy	The medicine of cost saving in three months^81^	US$22.10
	Public	Large	Surgery	A high-complexity surgical block savings^27^	7.40%
	Public	Medium	Operating room	Sterilization costs for a pediatric minor set^42^	17.50%
Inventory optimization	—	—	Supply rooms of NICU	Inventory optimization savings^82^	$17 452
	Private	Small	Pharmacy	Inventory cost^81^	49%
Participation in a bundled payment program	Public	All	Whole hospital	Lean IT adoption was found to be significantly related to participation in a bundled payment program^35^	OR = 2.060; *P* = .018
NPMAR	Public and private	All	Whole hospital	NPMAR was significantly correlated with lean^41^	b = 0.002, *P* < .05
VAR	Public	Large	Fertility clinic	VAR^18^	13%
RIE	Private	Large	Whole hospital	Mean annual benefit from that RIE^79^	$147 897

Abbreviations: IT, information technology; ED, Emergency department; NPMAR, net profit margin; NICU, neuro intensive care unit; VAR, Value-added ratio; RIE, rapid improvement events; OR, odds ratio. Notes: EBITDA = Earnings before interest, taxes, depreciation, and amortization margin. “—” means that there was no related information in the targeted articles. “All” means that hospital size included large, medium, and small. “-” means “negative sign.”

###  Theme 4: Satisfaction

 According to Lean’s core principles, both the concept of “patient first” and the principle of “respect for employees” are emphasized. This highlights Lean’s dual focus on improving outcomes for patients while valuing the contributions of healthcare staff. As such, the satisfaction metrics examined in this context include both patient satisfaction and hospital employee satisfaction. Based on, satisfaction in this study refers to the overall evaluation of the healthcare service process, service quality, interactions with care providers, and the work environment, as perceived by patients, healthcare providers, and other relevant personnel. By assessing experiences across multiple dimensions, it reflects the effectiveness and efficiency of healthcare services, as well as the degree to which the psychological and emotional needs of both patients and staff are addressed. Inductive analysis revealed several key indicators of satisfaction: patient satisfaction, Hospital Consumer Assessment of Healthcare Providers and Systems (HCAHPS) patient experience scores, complaint rates, and nurse satisfaction. We found that the studies in this dimension were predominantly conducted in public hospitals, large hospitals, or at the hospital-wide level. The departments involved included emergency (one studies), pharmacy (one study), and warehouse and logistics (one study). Seven studies were conducted at the hospital-wide level.

 A total of six studies investigated patient satisfaction, with five of them indicating an improvement in patient satisfaction linked to Lean implementation. One study, in particular, provided a detailed breakdown of satisfaction evaluation across various dimensions, including overproduction, waiting time, transportation, overprocessing, inventory, movement, and satisfaction defects.^83^ Regarding HCAHPS, which was primarily used in the United States to measure patient experience, three studies found a positive correlation between Lean implementation and improved patient experience scores.^35,40,76^ These findings suggest that Lean practices can enhance patient satisfaction and overall healthcare experience. Additionally, two studies examined the effect of Lean implementation by assessing changes in patient complaints, finding a reduction in complaint rates following Lean interventions.^20,60^ Only one study addressed nurse satisfaction, reporting a notable increase from 60.78% to 86.06% on the level of “very satisfied,”^71^ as summarized in Table 4.

**Table 4:** The Effect of Lean Applications in Hospitals on Satisfaction

**Codes**	**Hospital Type**	**Hospital Size**	**Departments**	**Indicators**	**Values**
Patient satisfaction	—	Small	Internal medicine, family medicine, and pediatric	Satisfaction included the adequacy of time spent with care providers during office visits, their care provider's ability to listen to their concerns and perceived staff helpfulness at the visit^56^	44.8 %, *P* < .05; 71.6%, *P* < .01; 55.4%, *P* < .01
	—	Large	Pharmacy	Overall satisfaction improved^57^	5.79 ± 3.61, *P* < .05
	Public	Large	ED	Overall satisfaction^39^	16%, *P* = .253
	Public	Medium	ED	Satisfaction of patients^60^	8.08%
	Public	—	Ear, nose, and throat	Patient satisfaction increased^40^	*P* < .05
	Private	Small	Inpatient	Inpatient satisfaction, including overproduction, waiting time, transportation, excess processing, inventory, motion, and satisfaction defects^83^	*P* = .019, *P* = .012; *P* = .011; *P* = .017; *P* = .010; *P* = .015; *P* = .010
HCAHPS patient experience scores	Public and private	All	Whole hospital	Hospital adoption of Lean was associated with higher HCAHPS patient experience scores and the degree of Lean implementation measured by the number of units throughout the hospital using Lean was associated with higher HCAHPS patient experience scores^76^	b = 3.35, *P* < .0001; b = 0.12, *P* < .012
	Public	All	Whole hospital	Lean adoption in public hospitals was marginally associated with HCAHPS patient experience ratings^40^	b = 0.114, *P* = .055
	Public	All	Whole hospital	Lean IT adoption was associated with a higher HCAHPS score^35^	b = 0.083, *P* = .051
Compliance rate	Public	—	Warehouse and logistics	The number of complaints^20^	-44.50%
	Public	Medium	ED	Compliance rate^60^	-4.85%, *P* < .001
Nurses’ satisfaction	Public	Large	Assessment	Nurses’ satisfaction^71^	25.28%, *P* < .0011

Abbreviations: ED, emergency department; HCAHPS, Hospital Consumer Assessment of Healthcare Providers and Systems. Notes: “—” means that there was no related information in the targeted articles. “All” means that hospital size included large, medium, and small. “-” means “negative sign.”

## Discussion

 The articles reviewed provide a comprehensive summary of the effects of Lean application in hospitals across four primary themes: efficiency, quality, cost, and satisfaction. Within these areas, the key contributions of Lean methodologies are effectively summarized, emphasizing its positive outcomes. The findings across the included articles consistently demonstrate the beneficial impact of Lean applications in healthcare settings. These positive results underscore the importance of promoting and further integrating Lean strategies in hospitals. Lean management, particularly focused on process optimization and waste reduction, offer valuable insights that can be applied to improve healthcare delivery, making them crucial for practitioners and policy-makers aiming to enhance hospital operations and patient care quality.

 Lean has been rapidly adopted since its introduction to the healthcare sector, particularly in developed countries, reflects its increasing recognition as a solution to improve operational efficiency. The United States, in particular, has been at the forefront of this movement, as evidenced by Antony et al,^2^ which reported that 47% of Lean-related research in hospitals originated from the US and the UK, with a further 23% from countries like Switzerland, Italy, and Brazil. Our findings were consistent with this pattern, showing that Lean is gaining substantial traction across various healthcare systems. Moreover, Lean implementation is steadily growing in developing countries, aligning with the observations made by Rathi et al.^84^ The expansion of Lean practices into developing regions indicates that these methodologies are increasingly regarded as a valuable tool for addressing healthcare challenges, even in resource-constrained settings. Lean implementation is a long-term process, and in many countries, especially developing ones, it is still in its early stages in healthcare. As a result, most studies demonstrate Lean effectiveness through case studies.^5,37,56,57^ Research evaluating entire hospitals is mainly led by the US,^24,40,76^ where Lean is more established, and specialized databases like the National Survey of Lean help assess Lean performance, facilitating healthcare research.

 We found that most lean initiatives were implemented in large public hospitals, with the ED, operating rooms, and pharmacies being the most commonly involved departments. This may be attributed to the fact that large public hospitals handle a higher volume of patients and complex medical processes, often with limited resources, which necessitates a greater focus on operational efficiency. Lean management can enhance operational efficiency and reduce costs by optimizing processes, eliminating waste, and ensuring the optimal allocation of resources.

 Lean management has long emphasized improving operational efficiency, and our research corroborated the widespread focus on this principle within the reviewed targeted articles. Specifically, Theme one, which pertains to efficiency improvement, was the most frequently addressed topic across the studies, with a total of 49 studies, accounting for 81.2% of the total targeted articles reviewed. This prevalence suggests that Lean implementation continues to be primarily examined through the lens of enhancing efficiency, reflecting the broader trend in healthcare management to prioritize resource optimization. Among the key efficiency factors examined, waiting times, LOS, and patient volume emerged as central themes in the application of Lean. These factors reflected areas that hospitals are currently prioritizing, likely because they represent the most accessible and impactful opportunities for improvement. Supporting our findings, a comprehensive review of Lean tools for healthcare process optimization by Barros et al^85^ similarly highlighted reductions in lead time, LOS, and costs as notable outcomes of Lean application. One significant point of divergence across the studies was the varying degree of reduction in waiting times, decreasing from 11.3%^5^ to 92.8%.^12^ These reported reductions in wait time also vary, mainly including bed hygiene waiting times,^12^ consultation waiting times,^5^ patient waiting times for prescriptions.^38^ These variations underscore the influence of context-specific factors on these indicators, such as the interventions used, hospital environments, and measurement methodologies. The heterogeneity in results is partly due to differing baseline comparisons (some studies used initial measurements, others tracked improvement rates), which introduces variability in both the assessment approach and the final outcomes. Our analysis demonstrated that Lean management was consistently effective in reducing hospital LOS,^12,24,35,40-43,61^ reinforcing the significant correlation between Lean adoption and reduced LOS. This finding illustrates how Lean strategies not only streamline operational processes but also enhance patient flow, contributing to better resource allocation and improved care delivery. Another notable finding in this review was the positive impact of Lean on patient volume. The implementation of Lean practices resulted in increased patient volumes, with improvements ranging from 15%^11^ to 68.9%,^5^ suggesting that Lean methodologies can optimize hospital throughput even within the constraints of existing resources. This suggests that Lean methodologies can enhance hospital efficiency by optimizing patient volumes, even within the constraints of existing resources. In addition, Lean interventions have also proven effective in other areas, such as facilitating patient discharged^16,40,43,44^; reduction in lead times,^20,37,45-47^ and shortening process times.^52-55^ These outcomes highlight Lean’s potential to improve hospital operations and efficiency, addressing both high-impact areas like patient flow and less obvious aspects such as discharge processes, demonstrating its versatility in healthcare.

 The second theme explored in this study was the impact of Lean implementation on quality improvement, a key concern for hospital practitioners. The 12 studies included in this review, though focused on different variables based on specific research objectives, all indicated a significant relationship between Lean practices and improvements in hospital quality. The most frequently examined variables were the 30-day readmission rates,^23,40,61,76^ counseling sessions,^47,52,65^ and drugs-related.^23,52,77^

 Three studies specifically identified a significant correlation between Lean implementation and reduced 30-day readmission rates, with one study reporting a decrease from 21% to 19.3% following Lean adoption.^61^ This suggests that Lean practices may enhance discharge planning and post-discharge care, potentially addressing common causes of readmission. Moreover, three studies highlighted that Lean resulted in increased patient consultation time and improvements in drug-related outcomes. These included better availability of pharmaceutical dosages and a reduction in the use of high-risk medications,^77^ as well as enhanced prescription verification procedures.^52^ These findings underscore Lean’s positive impact on patient safety, particularly in terms of medication management. Further, two studies from the United States documented a reduction in low-mortality DRGs,^47,71^ indicating that Lean may optimize hospital resource utilization, particularly for less critical cases. Another study observed a decrease in the incidence of pressure ulcers,^23^ further emphasizing Lean’s potential in improving patient outcomes in areas that require systematic monitoring and preventive measures. Although less frequently examined, variables such as patient safety,^40^ timeliness of care,^40^ and rational use of medical imaging^40^ were also addressed, reinforcing the broad applicability of Lean in enhancing multiple facets of hospital quality. By streamlining processes and fostering a culture of continuous improvement, Lean appears to address inefficiencies across various stages of patient care, thereby improving overall hospital performance. These findings suggest that Lean managements have significant potential to improve hospital quality across diverse domains, offering valuable insights for hospital managers seeking innovative solutions to enhance operational efficiency and quality care. Furthermore, the positive outcomes associated with Lean support its wider adoption in healthcare systems, with the potential to foster substantial improvements in both patient outcomes and operational efficiency.

 The third theme of this paper addressed the impact of Lean implementation on hospital costs. Studies focused on cost reduction, making this the second most frequently discussed topic after operational efficiency. This highlights the growing importance of cost reduction in contemporary hospital management, especially in response to global pressures to reduce healthcare spending while improving service efficiency. As a result, achieving cost reduction has become a central strategic goal for hospitals around the world. As Cegłowska et al noted in a review, lean management can positively influence treatment outcomes, which, in turn, can lead to cost reductions for healthcare systems.^86^ Our findings confirmed that Lean application can indeed help hospitals achieve cost savings. A review of Lean applications in Chinese hospitals reflected similar outcomes, though it also revealed that no hospital reported success in reducing patient care costs^4^. This suggests that while Lean can streamline operations and reduce overhead, its impact on direct care-related costs may be more nuanced and contingent on the specific organizational context. The most commonly reported areas of cost reduction include operating costs,^26,41,62,80^ inpatient expense per admission,^40,35,76^ and EBITDA.^24,35,40^ For instance, one study documented a 17.5% reduction in disinfection costs for pediatric minor sets,^49^ demonstrating Lean’s potential in optimizing non-clinical aspects of hospital operations. Given the diversity of the studies and the variation in the specific cost variables examined, this review offers a systematic perspective on the key cost-related variables commonly explored in Lean cost-reduction research. These variables served as critical indicators of Lean’s effectiveness in reducing healthcare costs, providing valuable insights for future research and practical application in hospital settings. The success of Lean in cost reduction depends not only on targeted processes but also on organizational culture and commitment to continuous improvement. Hospitals that effectively implement Lean typically foster a collaborative culture, with staff at all levels engaged in problem-solving and process redesign.

 The final theme discussed in this paper was satisfaction, a critical yet underexplored area in Lean research. Although Lean principles emphasize the significance of improving both patient and staff experience to enhance overall satisfaction, these comprehensive variables are often overlooked in studies, as project stakeholders tended to focus more on the tangible outcomes of Lean implementation. Our analysis revealed that most studies on satisfaction focused on patient satisfaction,^39,47,56,57,60,83^ with six studies included in this theme. Talero-Sarmiento also highlighted a significant body of literature focused on adopting Lean strategies to improve patient satisfaction.^87^ Furthermore, two reviews reported that lean management was positively associated with their job satisfaction.^88,89^ In contrast, only one study examined nurse satisfaction.^71^ An interesting novel finding from this review was that two articles mentioned a decrease in complaint rates after Lean implementation, which indirectly reflects an increase in satisfaction.^20,60^ Additionally, HCAHPS scores, a key metric for assessing patient satisfaction in US hospitals, were frequently discussed across studies.^35,40,76^ In contrast, other countries often rely on more traditional post-implementation satisfaction scales. These insights highlight the need for a more balanced approach to Lean research, incorporating both patient and staff satisfaction.

 This approach is essential for achieving sustained improvements in healthcare delivery, as the long-term success of Lean initiatives depends not only on operational efficiencies but also on the well-being and engagement of those involved in patient care. Integrating staff satisfaction metrics, particularly for nurses, offers a more holistic view of Lean’s impact, enhancing our understanding of its potential to improve healthcare quality and sustainability.

 This review examines the positive effects of Lean implementation in hospitals from four distinct dimensions, providing a fresh perspective that aligns with the current priorities of healthcare institutions. We intended to offer valuable insights for hospital administrators and policy-makers when considering the introduction or application of Lean management. However, this study had its limitations. First, the scope of the search was restricted to recent years, thereby limiting the selection of relevant literature. Second, although we considered lean-related terminology, there may still be cases where some terms are missing, and relevant literature could be overlooked.

 Third, while all the included studies report positive outcomes from Lean implementation, the majority focus on single departments or specific projects, which restricts the ability to draw definitive causal conclusions. Fourth, considerable variation in the terminology, tools, and methods used across the targeted articles makes it challenging to identify a standardized context for Lean application, and consequently, the review could only provide a broad summary of the key areas in which improvements were observed.

 Future research should examine long-term effects of lean application in hospitals across diverse hospitals, particularly in developing countries. This will help ensure the broader applicability and sustainability of Lean practices in a variety of healthcare environments, facilitating continued improvements in patient care, operational efficiency, and financial viability.

## Conclusions

 This review synthesizes the effects of Lean management in healthcare, focusing on four key themes: Efficiency, quality, cost, and satisfaction. We found that most hospitals adopting Lean are large public hospitals, particularly in high-impact departments such as emergency rooms, operating rooms, and pharmacies. These departments, with high patient volumes and complex processes, benefit most from Lean to streamline operations and reduce costs. Key efficiency improvements include reduced waiting times, shorter LOS, and better patient flow, all contribute to enhanced resource utilization. Lean also drives significant quality improvements, such as lower 30-day readmission rates, improved medication management, and heightened patient safety. These outcomes highlight the benefits of Lean to improve patient care through better discharge planning, consultations, and safer medication practices. Financially, Lean management results in cost reductions by enhancing treatment outcomes and operational efficiency, which is crucial in the current global healthcare landscape focused on cost containment. By optimizing processes and reducing waste, Lean fosters both improved hospital performance and long-term financial sustainability. Regarding satisfaction, most studies focus on patient satisfaction, with fewer addressing employee satisfaction. Patient satisfaction was also evaluated through compliance rates. Future research should explore Lean’s long-term effects in diverse hospital environments, particularly in developing countries, to ensure broader applicability and sustainability.

## Practice Implications

 The application of Lean in hospitals offers significant value across various domains, benefiting hospitals, healthcare managers, and policy-makers. Studies consistently highlight the benefits from Lean management, particularly in improving operational efficiency in large hospitals. Lean management is helpful to create a more efficient and resource-effective environment by reduce waiting times, LOS, and optimize patient flow. Lean also enhances both clinical and non-clinical outcomes, such as lowering operating costs, inpatient expenses, and improving resource allocation (eg, cost reductions in pediatric department disinfection). These efficiencies support profitability while ensuring effective resource use, crucial for hospitals with limited budgets. For healthcare managers, Lean management provides a framework to improve both patient care quality and operational efficiency. Lean consistently lowers 30-day readmission rates, enhances patient safety, and addresses medication-related issues, especially in improving post-discharge care. These improvements aid in reducing costly readmissions and enhance care continuity. Additionally, Lean fosters greater staff engagement and job satisfaction, encouraging a culture of continuous improvement and operational excellence. For policy-makers, Lean practices offer a solution to controlling healthcare costs while maintaining or improving service quality. Policy-makers can leverage Lean to enhance patient flow, minimize unnecessary procedures, and optimize care delivery, thus meeting the growing demand for cost-effective, patient-centered healthcare systems.

## Acknowledgements

 The authors acknowledge the time and effort that the participants devoted to this review. Additionally, we would like to thank the Second Affiliated Hospital of Henan Medical University for their financial support in the publication of this review.

## Ethical issues

 This study does not involve human participants, human data, or any animal studies. Therefore, ethical approval was not required.

## Conflicts of interest

 Authors declare that they have no conflicts of interest.

## Supplementary files


Supplementary file 1. The Full Search Strategies in This Review.

Supplementary file 2. Risk of Bias for Articles Included in This Review.
