# Supplementary material for: A Systematic Review of Lean Implementation in Hospitals: Impact on Efficiency, Quality, Cost, and Satisfaction
Source: Int J Health Policy Manag. 2025 Aug 26;14:8974. doi: 10.34172/ijhpm.8974 (PMC12573144; doi:10.34172/ijhpm.8974)
Supplement: Supplementary file 1 — The Full Search Strategies in This Review. [file ijhpm-14-8974-s001.pdf]

Article title: A Systematic Review of Lean Implementation in Hospitals: Impact on Efficiency, Quality, Cost, and Satisfaction

Journal name: International Journal of Health Policy and Management (IJHPM)

Authors' information: Jingjing Wang[1,2], Hui Lv[3], Mingxin Chen[4], Chenyang Liu[4], Wenjie Ren[1]*, Hui Jiang[1], Lizhang Zhang[1]

[1]Institutes of Health Central Plains, Henan Medical University, Xinxiang, China.

[2]The Second Affiliated Hospital of Henan Medical University, Xinxiang, China.

[3]The First Affiliated Hospital of Henan Medical University, Xinxiang, China.

[4]School of Public Health, Henan Medical University, Xinxiang, China.

*Correspondence to: Wenjie Ren; Email: rwj1571373@126.com

Citation: Wang J, Lv H, Chen M, et al. A systematic review of lean implementation in hospitals: impact on efficiency, quality, cost, and satisfaction. Int J Health Policy Manag. 2025;14:8974. doi:10.34172/ijhpm.8974

Supplementary file 1. The Full Search Strategies in This Review

Source	Search Terms	Results
PubMed	1: ((((((((((Lean management) OR (Lean principles)) OR (Lean thinking)) OR (Lean approach)) OR (Lean healthcare)) OR (Lean health care)) OR (Lean production)) OR (Lean process)) OR (Lean culture)) OR (Lean methods)) OR (Lean methodology) Filters: from 2019/1/1 - 2024/10/31<br>2: ((((Hospital) OR (Healthcare sector)) OR (Healthcare service)) OR (Medical institutions)) OR (Hospital setting) Filters: from 2019/1/1 - 2024/10/31<br>3: ((Effectiveness)OR (Efficiency)) OR (Quality) OR (Cost) OR (Satisfaction) Filters: from 2019/1/1 - 2024/10/31<br>4: #1 AND #2 AND #3	3081
Scopus	((TITLE-ABS-KEY (lean AND management) OR TITLE-ABS-KEY (lean AND principles) OR TITLE-ABS-KEY (lean AND thinking) OR TITLE-ABS-KEY (lean AND approach) OR TITLE-ABS-KEY (lean AND healthcare) OR TITLE-ABS-KEY (lean AND health AND care) OR TITLE-ABS-KEY (lean AND production) OR TITLE-ABS-KEY (lean AND process) OR TITLE-ABS-KEY (lean AND culture) OR TITLE-ABS-KEY (lean AND methods) OR TITLE-ABS-KEY (lean AND methodology)) AND PUBYEAR > 2018 AND PUBYEAR < 2025) AND ((TITLE-ABS-KEY (hospital) OR TITLE-ABS-KEY (healthcare AND sector) OR TITLE-ABS-KEY (healthcare AND service) OR TITLE-ABS-KEY (medical AND institutions) OR TITLE-ABS-KEY (hospital AND setting)) AND PUBYEAR > 2018 AND PUBYEAR < 2025) AND ((TITLE-ABS-KEY (effectiveness) OR TITLE-ABS-KEY (efficiency) OR TITLE-ABS-KEY (quality) OR TITLE-ABS-KEY (cost)) OR TITLE-ABS-KEY (satisfaction)) AND PUBYEAR > 2018 AND PUBYEAR < 2025)	1033
Web of Science	1: ((((((((((TS=(Lean management)) OR TS=(Lean principles)) OR TS=(Lean thinking)) OR TS=(Lean approach)) OR TS=(Lean healthcare)) OR TS=(Lean health care)) OR TS=(Lean production)) OR TS=(Lean process)) OR TS=(Lean culture)) OR TS=(Lean methods)) OR TS=(Lean methodology) and Preprint Citation Index (Exclude – Database)<br>((((TS=(Hospital)) OR TS= (Healthcare sector)) OR TS= (Healthcare service)) OR TS= (Medical institutions)) OR TS= (Hospital setting) and Preprint Citation Index (Exclude – Database)<br>3: ((((TS=(Effectiveness)) OR TS=(Efficiency)) OR TS=(Quality)) OR TS=(cost))) OR TS=(Satisfaction) and Preprint Citation Index (Exclude – Database)<br>4: #1 AND #2 AND #3 and Preprint Citation Index (Exclude – Database)	1013
Medline	S1: Lean management OR Lean principles OR Lean thinking OR Lean approach OR Lean healthcare OR Lean health care OR Lean production OR Lean process OR Lean culture OR Lean methods OR Lean methodology<br>S2: Hospital OR Healthcare sector OR Healthcare service OR Medical institutions OR Hospital setting<br>S3: Effectiveness OR Efficiency OR Quality OR Cost OR Satisfaction<br>S4: S1 AND S2 AND S3	859
