# Supplementary material for: A Systematic Review of Lean Implementation in Hospitals: Impact on Efficiency, Quality, Cost, and Satisfaction
Source: Int J Health Policy Manag. 2025 Aug 26;14:8974. doi: 10.34172/ijhpm.8974 (PMC12573144; doi:10.34172/ijhpm.8974)
Supplement: Supplementary file 2 — Risk of Bias for Articles Included in This Review. [file ijhpm-14-8974-s002.pdf]

Article title: A Systematic Review of Lean Implementation in Hospitals: Impact on Efficiency, Quality, Cost, and Satisfaction

Journal name: International Journal of Health Policy and Management (IJHPM)

Authors' information: Jingjing Wang[1,2], Hui Lv[3], Mingxin Chen[4], Chenyang Liu[4], Wenjie Ren[1]*, Hui Jiang[1], Lizhang Zhang[1]

[1]Institutes of Health Central Plains, Henan Medical University, Xinxiang, China.

[2]The Second Affiliated Hospital of Henan Medical University, Xinxiang, China.

[3]The First Affiliated Hospital of Henan Medical University, Xinxiang, China.

[4]School of Public Health, Henan Medical University, Xinxiang, China.

*Correspondence to: Wenjie Ren; Email: rwj1571373@126.com

Citation: Wang J, Lv H, Chen M, et al. A systematic review of lean implementation in hospitals: impact on efficiency, quality, cost, and satisfaction. Int J Health Policy Manag. 2025;14:8974. doi:10.34172/ijhpm.8974

Supplementary file 2. Risk of Bias for Articles Included in This Review

No.	Authors(year)	Abstract and title	Introduction and aims	Method and data	Sampling	Data analysis	Ethics and bias	Results	Transferability/ Generalizability	Implications and usefulness	Total score	Levels
1	Hung et al. (2021)	4	3	4	4	4	1	3	2	3	28	Medium
2	Muharam and Firman (2022)	4	3	4	4	4	4	4	2	3	32	High
3	Martín-Conde et al. (2021)	3	2	3	3	4	1	2	2	2	22	Low
4	Sales-Coll et al. (2021)	3	4	4	3	2	1	4	2	3	26	Medium
5	Reis et al.(2023)	4	4	3	3	2	1	3	3	4	27	Medium
6	Drei and Ignacio (2022)	4	3	3	3	2	1	2	3	3	24	Medium
7	Rocha and Vasconcelos (2024)	4	3	3	3	2	1	2	3	3	24	Medium
8	Bossone et al. (2022)	4	4	3	3	3	4	4	3	3	31	High
9	Ullah et al. (2020)	4	2	4	2	4	4	4	3	3	30	High
10	Zhang et al. (2021)	4	3	4	3	4	1	4	3	3	29	Medium
11	Hammoudeh et al. (2021)	4	3	4	4	4	4	4	3	3	33	High
12	Cotton et al. (2020)	3	3	3	2	2	1	2	3	3	22	Low
13	Po et al. (2019)	4	4	4	4	4	1	4	4	4	33	High
14	Roey et al.(2023)	3	4	4	4	4	1	4	4	4	32	High
15	McWilliams et al. (2019)	3	3	3	3	2	1	2	3	3	23	Medium
16	Zhang et al. (2021)	4	4	4	3	4	1	4	3	3	30	High
17	Mangum et al. (2021)	4	2	3	2	2	1	3	3	3	23	Medium
18	Coll et al. (2021)	4	3	3	2	2	1	2	3	3	23	Medium
19	Fiorillo et al. (2021)	4	4	4	3	3	1	4	3	3	29	Medium
20	Lee et al. (2024)	4	3	4	3	4	1	4	4	4	31	High
21	Alexander et al. (2019)	3	2	4	4	4	4	3	3	3	30	High
22	Sales et al. (2023)	4	3	3	2	2	1	2	3	3	23	Medium
23	Freitas et al. (2023)	3	4	4	3	4	1	4	3	3	29	Medium
24	Verolino et al. (2021)	4	3	4	3	4	4	4	3	4	33	High
25	Rollinson et al. (2021)	3	4	4	4	2	4	2	3	3	29	Medium

26	AlHarthy et al. (2023)	4	4	3	4	4	4	4	4	4	35	High
27	Feinman et al. (2022)	3	2	3	3	2	1	3	2	3	22	Low
28	Yuliati and Andriani (2021)	4	3	3	4	2	1	2	3	3	25	Medium
29	Kurnia et al. (2023)	4	3	3	3	2	1	2	3	2	23	Medium
30	Rizan et al. (2020)	4	4	3	3	2	1	2	3	4	26	Medium
31	Lin et al. (2023)	4	3	4	3	4	4	4	3	3	32	High
32	Alzahrani (2020)	4	4	4	4	3	1	2	3	4	29	Medium
33	Sallam et al. (2024)	4	3	4	3	3	4	3	3	4	31	High
34	Trakulsunti et al. (2022)	4	4	3	2	2	1	2	3	4	25	Medium
35	Meyer et al. (2020)	3	3	3	2	2	1	3	3	3	23	Medium
36	Papp et al. (2021)	4	3	4	4	4	1	3	3	4	30	High
37	Ibrahim et al. (2019)	4	3	3	3	4	4	4	3	4	32	High
38	Cerfolio et al. (2019)	4	3	3	3	4	1	4	3	3	28	Medium
39	Ankrum et al. (2019)	4	2	4	3	4	4	4	3	4	32	High
40	Letelier et al. (2021)	4	3	3	4	4	1	4	2	3	28	Medium
41	Epistola et al. (2023)	4	2	3	3	3	1	3	2	3	24	Medium
42	Herlihy et al. (2023)	4	3	3	3	3	1	3	3	4	27	Medium
43	Boyle et al. (2022)	4	2	3	2	4	4	4	2	3	28	Medium
44	Goretti et al. (2023)	4	4	4	3	3	1	3	3	4	29	Medium
45	Wang et al. (2023)	4	3	4	4	4	4	4	3	4	34	High
46	Balcom and Reyes (2019)	3	3	3	2	3	1	2	2	3	22	Low
47	Rachh et al. (2023)	3	3	4	3	3	1	3	3	3	26	Medium
48	Li et al.(2023)	4	4	4	4	4	4	4	3	4	35	High
49	Ilangakoon et al. (2022)	4	4	4	4	4	1	4	3	4	32	High
50	Rundall et al. (2021)	4	4	4	3	4	1	4	4	4	32	High
51	Shortell et al. (2021)	4	4	4	3	4	1	4	3	4	31	High
52	Gayoso-Rey et al. (2020)	3	3	3	3	3	1	3	4	4	27	Medium
53	Tierney et al. (2021)	4	3	3	3	3	1	3	3	3	26	Medium
54	Ayaad et al. (2022)	4	4	4	4	4	4	4	3	4	35	High

55	Menachemi et al. (2020)	3	3	4	4	4	1	4	4	4	31	High
56	Souza et al. (2020)	4	4	4	3	3	1	3	3	3	28	Medium
57	Abdallah (2020)	3	4	4	4	4	1	4	3	4	31	High
58	Iswanto and Rosady (2020)	3	4	3	4	3	1	3	3	3	27	Medium
59	Yaglowski (2024)	2	3	3	3	2	1	2	3	3	22	Low
60	Harahap et al.(2023)	4	4	3	4	4	1	4	3	4	31	High
